# A qualitative study of women’s network social support and facility delivery in rural Ghana

**DOI:** 10.1371/journal.pone.0206429

**Published:** 2018-11-06

**Authors:** Leslie E. Cofie, Clare Barrington, Sodzi Sodzi-Tettey, Susan Ennett, Suzzane Maman, Kavita Singh

**Affiliations:** 1 Department of Health Education and Promotion, East Carolina University, Greenville, United States of America; 2 Department of Health Behavior, University of North Carolina, Gillings School of Global Public Health, United States of America; 3 Carolina Population Center, University of North Carolina, United States of America; 4 Institute for Healthcare Improvement, Accra, Ghana; 5 Department of Maternal and Child Health, University of North Carolina, Gillings, School of Global Public Health, United States of America; University of Michigan Medical School, UNITED STATES

## Abstract

Similar to many sub-Saharan African countries, maternal mortality in Ghana ranks among the highest (39^th^) globally. Prior research has demonstrated the impact of social network characteristics on health facility delivery in sub-Saharan Africa. However, in-depth examination of the function of all members in a woman’s network, in providing various types of support for the woman’s pregnancy and related care, is limited. We qualitatively explore how women’s network social support influences facility delivery. Qualitative data came from a mixed methods evaluation of a Maternal and Newborn Health Referral project in Ghana. In 2015 we conducted in-depth interviews with mothers (n = 40) and husbands (n = 20), and 4 focus group interviews with mothers-in-law. Data were analyzed using narrative summaries and thematic coding procedures to first examine women’s network composition during their pregnancy and childbirth experiences. We then compared those who had homebirths versus facility births on how network social support influenced their place of childbirth. Various network members were involved in providing women with social support. We found differences in how informational and instrumental support impacted women’s place of childbirth. Network members of women who had facility delivery mobilized resources to support women’s facility delivery. Among women who had homebirth but their network members advocated for them to have facility delivery, members delayed making arrangements for the women’s facility delivery. Women who had homebirth, and their network members advocated homebirth, received support to give birth at home. Network support for women’s pregnancy-related care affects their place of childbirth. Hence, maternal health interventions must develop strategies to prioritize informational and instrumental support for facility-based pregnancy and delivery care.

## Introduction

Health facility delivery with adequate quality of care is one of the most efficient and cost-effective ways to reduce maternal mortality [1, 2]. Births outside of health facilities can result in maternal death from pregnancy-related complications such as hypertensive disorder, postpartum hemorrhaging, sepsis, and complications from childbirth [3]. Sub-Saharan African countries account for approximately 62% of the world’s maternal mortality, and over half of all births in this region occur outside of health facilities [4]. Compared to some sub-Saharan African countries, the maternal mortality ratio (MMR) in Ghana is lower at 319 per 100 000 live birth. This ratio is ranked among the highest (39^th^) globally, and thus Ghana remains off track to achieve the third sustainability development goal of reducing maternal deaths to less than 70 per 100 000 live births by the year 2030 [4–6]. According to the 2014 Ghana Demographic Health Survey (GDHS) 73% of births in Ghana occurred in health facilities, whereas only 59% of births in rural areas were in facilities [7]. Further improvement in uptake of facility delivery is needed, as more than half of Ghana’s population lives in rural settings.

Determinants of health facility delivery among women in Africa include maternal socio-demographic, economic, physical access and facility-related factors health provider attitude, privacy, and quality of care [8, 9]. In Ghana, affordable pregnancy-related care, short distance and accessible transportation to a health facility are associated with facility births [10–12]. Maternal characteristics such as older age, higher education level, perceived high quality of health care and treatment received from health providers are also associated with facility delivery [12–15].

There is some evidence of community level determinants of facility delivery including community attitudes, social norms and social networks have been little studied. Researchers have also highlighted the need for studies to examine how determinants like social networks may contribute to uptake in women’s use of facility delivery care [2, 8]. Investigating such determinants may provide insights into additional areas of interventions to increase uptake in facility delivery [2, 8, 16]. For instance, women integrated into a social network receive information and resources diffused through their network members that may be pertinent to their use of maternal health services [17, 18].

Social networks are relevant for examining ways in which social relations influence health [19]. A social network is a web of social relationships among individuals that has both structural and functional characteristics [20, 21]. Network structure refers to the properties of the relationships between an individual and others in his/her network [22, 23]. It includes network composition, which describes the types of individuals in a network (e.g. relatives, neighbors and friends) and the attributes of these individuals [24, 25]. Network functions include exchange of social influence and social support among network members [26, 27]. Social support can be beneficial for promoting individual wellbeing by operating as the means in which networks members act to influence the individual’s mental and physical health [19].

Support may be in the form of informational (advice and suggestions), instrumental (aid or assistance), and emotional (empathy, care and trust) support received from network members [28]. Past qualitative studies found an association between social support and women’s use of facility delivery care [29–31]. These studies explored support including instrumental and information support provided by one or two specific network members, or from women’s own perspective, support received that was relevant for accessing facility delivery. However, women’s perception of the extent to which all members in their network provided some form of support that impacted their pregnancy experiences and place of birth were not examined.

For example, Story et al., [29] explored husbands’ involvement in women’s use of facility delivery in rural Bangladesh and found that women whose husbands provided them with informational, emotional and instrumental support were more likely to experience facility births, compared with those whose husbands were unsupportive. Recent evidence from Ghana also suggests that women were dependent on network members for instrumental support (economic or logistics) to get them to a health facility for childbirth [30]. Whereas the types of support women receive may be dependent on their network members’ competing or unifying interests in their pregnancy experience and place of delivery [32], the available studies did not examine the complete networks of women. Addressing this gap in the literature will provide critical insights into how network members operate as a whole to provide support for women.

Knowledge of the network support roles of all members in a woman’s network, could significantly underscore the benefit of incorporating social networks in intervention strategies to increase uptake in facility delivery in Ghana [2, 8, 16]. To that end, our study examines the social network dynamics of all members of women’s social networks during pregnancy and childbirth, by characterizing the social network dynamics of women in rural Ghana. We then analyze qualitative interviews with mothers, fathers, and mothers-in-law in order to provide in-depth description of women’s network provision of social support. Also, we compare women who had facility birth and homebirths, on how network support influenced their place of childbirth.

### Study setting

This study was based in the Northern region, located in the northern part of Ghana; and the Central region in the southern part [33]. The estimated total populations in these regions at 2,201,863 and 2,479,461 respectively [33]. The proportion of the rural population in the Northern region (NR) and Central region (CR) are approximately 70% and 53% respectively [33]. The CR had a MMR of 520 maternal deaths per 100,000 live births and the NR had a MMR of 531, per the 2014 GDHS [7]. The proportion of health facility delivery in the NR (35%) is lowest among all regions in Ghana, whereas the proportion in the CR (70%) is lower than the national proportion [7].

Most households in rural communities in both regions are considered poor, and few have access to a local health facility such as clinics, health post, or Community-Based Health Planning Services Compound that may be understaffed and lacking equipment [7]. These facilities are often understaffed, lack pregnancy related care equipment, have limited communication services and lack transport systems. Most people have to travel miles to access higher-level health facilities such as health centers and hospitals. In the NR, women rely on their family members and sometimes their neighbors to secure transportation to access health facilities [34]. In the Central region transportation is accessible on main roads, although this can be costly and not necessarily reliable [34, 35]. Women who live in very remote villages have to walk miles to get to a main road to find transportation.

Males are heads of 85% of households in the NR and 60% of households in the NR [33]. The average household size is 8 in the NR, and 4 in the CR. However, in both regions households tend to be clustered in close-knit communities in rural areas, with extended family members living nearby. Although traditional religious practices are common on both regions, Islam is the predominant religion in the NR (60%) and Christianity is predominant in the CR, 83% [33]. Polygamy is practiced in the NR. Traditionally, NR women move from their own community to live in their husband’s after marriage. Therefore, women often have very little interaction with their blood relatives after marriage. Also, it is customary for husbands and mothers-in-laws to make decisions about women’s pregnancy related health needs and to subsequently provide them with resources for pregnancy and delivery care in the NR. Notably, fathers-in-law play a significant role as compound heads in authorizing the utilization of these resources to care for pregnant women. Conversely, CR women often do not move away from their community after marriage, and maintain close interactions with their blood-relatives. Husbands, mothers and mothers-in-law in this region make decision about women’s pregnancy related care, often with the women’s input.

## Methods

### Design and sample

**Fig 1:**
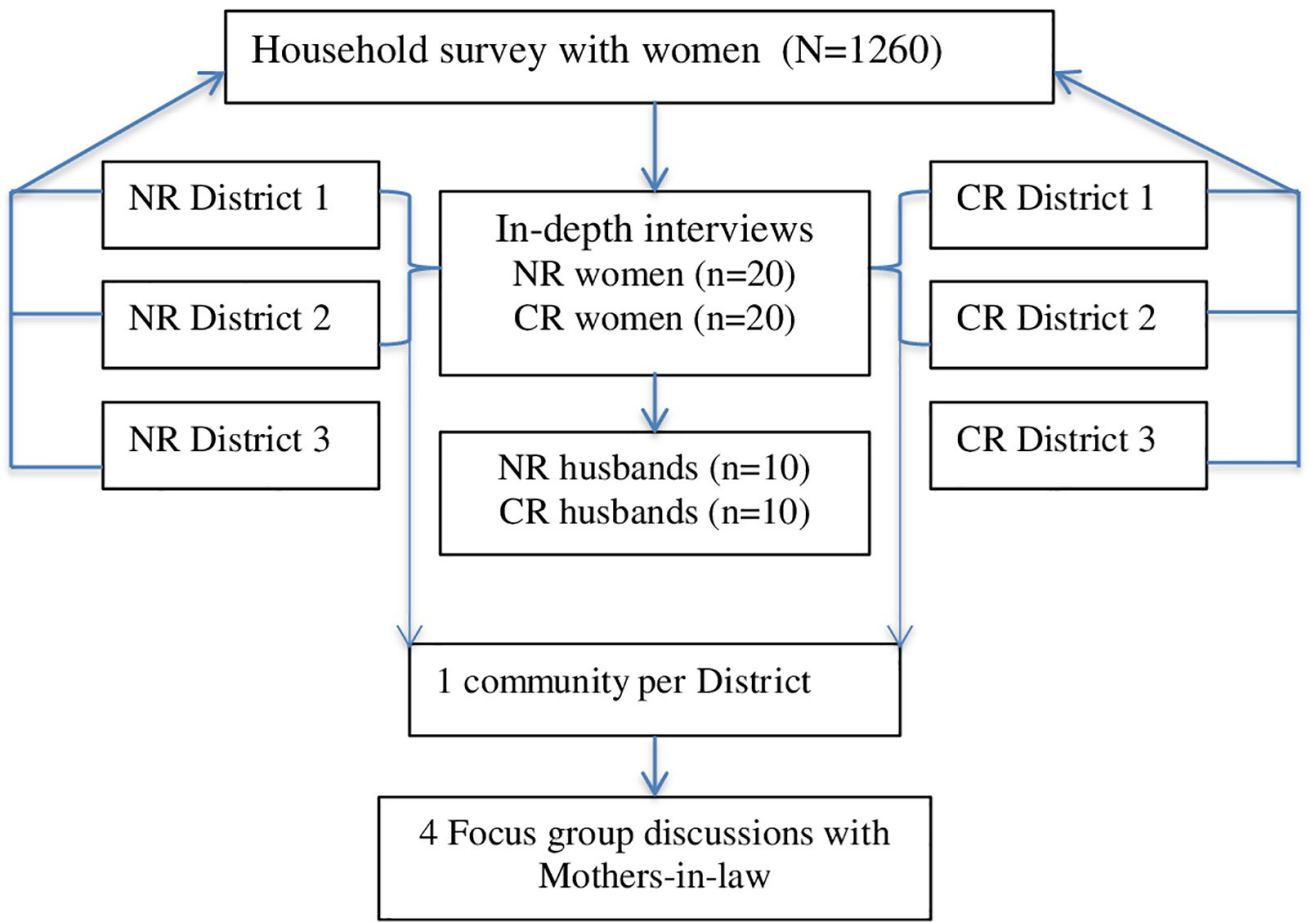
Study design and sample description.

This study was approved by the Ghana Health Service Ethical Review Committee and exempted from ethics review by University of North Carolina (UNC)–Chapel Hill’s Internal Review Board, as it was considered a program evaluation. Data for the study came from a mixed methods evaluation of an intervention to improve access to maternal and newborn health services and outcomes in rural Ghana by strengthening the referral process for pregnant women and sick newborns with complications. Between January and March 2015, household surveys were administered to 1260 women of reproductive age in 3 districts each in the NR and CR. We recruited a subsample of women who participated in the household surveys to participate in individual qualitative interviews (Fig 1).

In-depth interviews (IDIs) were conducted with 20 women from across 2 randomly selected districts each in the NR and CR. Also, a sample of the women’s husbands (10 each in the NR and CR) was interviewed. Four focus group discussions (FGDs) were conducted with mothers-in-law (MILs) (8 to 12 per group) who were selected from a community in each of the district where the IDIs were conducted. We purposively sampled women who gave birth, and MILs (unrelated to the women selected for the IDIs) whose daughters-in-law gave birth, in the past 12 months prior to data collection. To enable comparison on use of facility delivery, we also sampled equal numbers of women who had facility births and homebirths. Two interviews each with NR and CR women, and one interview with a NR husband were lost, due to data corrupted audio files (Table 1).

**Table 1:** Sample size of participants.

		North (N) 2 Districts: ~15 communities	Central (N) 2 Districts: ~15 communities	Total (N)
**Qualitative methods:** *Individual interview*	Women	18	18	36
	Husbands	9	10	19
	MILs	2	2	4

### Data collection

We used an egocentric network approach to collect qualitative data on women’s social network composition and function [36]. This approach enables examination of network characteristics from the women’s (focal individuals) perspectives, rather than interviewing all members of their networks. Women’s perceptions of their social networks are well correlated with the actual attributes of their network members, and thus serve as a reliable indicator of their network members’ characteristics [37]. To assess network composition, women were asked to first provide a comprehensive list of all network members involved in their pregnancy experiences. They then provided information on how they are related, and their frequency of contact and interactions with the network members; how long they have known the members; and how far network members live from them. Women also described the type of relationship they have with network members and which network members know and interact with each other. To assess network function, women were asked about how each network member was involved in their pregnancy experiences and the kinds of support and influence each member provided on their health service utilization during pregnancy and delivery.

Husbands were asked to describe their wives’ pregnancy experiences, identify roles of individuals involved in health decisions about their pregnancy, and describe the kinds of support they and other family members provided their wives. In FGDs MILs were asked to identify roles of individuals involved in health decisions about women’s pregnancy, describe community perceptions about place of childbirth, and describe various supports MILs and family members provide women.

Our objective was to understand important themes related to network support and women’s pregnancy and delivery experiences. During fieldwork the first author (LC) met with research assistants at the end of each day to review their summaries of the day’s interviews and to discuss emerging themes. Data saturation, the point at which additional data did not yield new insights on key themes relevant to the study, was reached before completing the interviews with women and their husbands [38]. However, the sample size was not modified because this study was part of a larger research study that covered other topics of relevance to the overall study (Table 1). Two additional FGDs with mothers-in-law were added in order to reach saturation for the FGDs.

In each study region, male and female research assistants who were experienced in conducting qualitative data collection in the local languages of the study communities (*Twi* and *Fanti* in the CR and *Dagbani* and *Lekpepkel* in the NR) conducted the interviews and took detailed field notes. The first author trained them to collect qualitative social network data. Research assistants obtained informed verbal consent from all study participants, and also verbal consent from guardians of women under the age of 18 years. They read aloud informed consent forms to study participants, and guardians when appropriate. The research assistants then noted on the forms when verbal consents were received. Ethics review approval for the consent process was obtained from the Ghana Health Service and the UNC-Chapel Hill. All interviews were audio-recorded and transcribed into English.

### Data analysis

Our analysis began with close readings of all IDI and FGD transcripts. LEC wrote narrative summaries of pregnancy and delivery experiences, and of each FGD with MILs on their perceptions about childbirth experiences of women in their communities. LEC, with input from CB, then generated preliminary descriptive codes and memos on the various roles of network members in women’s pregnancy and delivery care experiences using these summaries. After another review of the transcripts and the research team’s discussion of emergent themes from the narrative summaries and field notes, LEC developed a core set of inductive codes in order to conduct thematic analysis of women’s social network characteristics and pregnancy/delivery care experiences. LEC applied these codes to the IDIs and FGDs using Atlas.ti software (version 7.0, Scientific Software Development GmbH, Eden Prairie, MN), and with input from CB modified and added new codes during the coding process [39]. After coding all transcripts, LEC conducted coding checks by reviewing all the transcripts to ensure that the coded data reflected codes defined in the codebook. Discrepancies identified were discussed with CB corrected to ensure coding accuracy and consistency.

Subsequently, LEC worked with the research team in analyzing and interpreting that data. We reviewed the code outputs and developed code summaries and analytic matrices [40, 41]. We used both code summaries and narrative summaries, which captured women’s interactions with network members during pregnancy, to create a table summarizing women’s network composition, and described contextual information on the supportive roles of network members. In the matrices, we compared women who had facility versus homebirth and NR versus CR women, on themes of how network support in women’s receipt of pregnancy, labor, and delivery care affected their place of delivery.

## Results

We compared network members’ intention for women to use either facility or homebirth and where women actually gave birth, and identified three mutually exclusive groups of women. The facility birth group, included women who had facility deliveries and all had network members that advocated for them to have facility birth. Second, was the unintended-homebirth group, which included women who had homebirth but indicated that their network members advocated for them to have facility birth. The third group, the homebirth group, included women who had homebirth and had network members who advocated for them to have home delivery. We first describe the study population. Next, we examine differences in how network intentions and provision of different kinds of support might explain women’s place of childbirth.

### Study population characteristics

Overall, 17 women gave birth in a health facility and 19 gave birth at home (Table 2). The average ages of women and husbands were 27 and 36 years, respectively. All NR women were married, most had no formal education and were farmers by occupation. Most NR women had co-wives. Only 2 CR women were unmarried, most had at least a junior secondary (junior-high) school level education and were farmers. All women provided a comprehensive list of network members involved in their pregnancy experiences. Women’s network size was on average 8 people. All women had blood-relatives and in-laws in their networks; about two-thirds of women’s network also included at least one friend. Generally, family members made up more than half of each woman’s network.

**Table 2:** Demographic characteristics of women and husbands.

		North (N)				Central (N)				Total (N)			
**Age** (Max| *mean*| Min)	Women	40	*26*		20	38	*27*		17	40	*27*		17
	Husbands	58	*35*		26	62	*37*		23	62	*36*		23
**Married**		18				16				33			
**Education** (Women | Husbands)	None	11		5						11		5	
	Primary	3		1		5		2		8		3	
	JSS	5		1		13		3		18		4	
	SSS			1				5				6	
	A level			1								1	
**Occupation** (Women | Husbands)	Farmer	15		7		5		5		20		12	
	Trader/seller					6				6			
	Skilled worker	3		2		2		5		5		7	
	Unemployed					5		1		5		1	
**Religion** (Women | Husbands)	Moslem	8		1						8		1	
	Christian	5		2		17		10		22		12	
	Traditionalist	4		6						4		6	
**Place of delivery**	Facility birth	8				9				17			
	Homebirth	10				9				19			

We compared network support by region, and across the different groups of women (facility birth, unintended-homebirth, and homebirth groups) to determine if network function could help explain the different outcomes in terms of place of delivery. As we did not observe any meaningful differences by region, we subsequently examined social support received by women across the three groups and found some differences in informational and instrumental social support by group.

### Network support of women’s pregnancy and birth delivery experience

Generally, all women received some form of emotional, informational and instrumental support from their network members during pregnancy (Table 3).

**Table 3:** Social support and network composition among three groups of women that had either health facility of homebirth.

Dynamics of network relationships	Groups of women	Emotional support	Informational support	Instrumental support
Similar across network support patterns: *Network proximity*–most network members lived in the same house with women, or nearby *Frequency of contact*–most members regularly visited with women (at least a few times daily *Nature of relationships*–women general had “good” and or “close” relationships with network members Most members in women’s network knew and had contact with each other	***Facility birth*** Network members intended to facilitate women’s facility delivery, and actually did so (n = 17)	Across network support patterns most women indicated that network members were caring/ showed empathy in their interactions with them	Most women (n = 11) received advice to use facility-based pregnancy and or delivery care	Network members worked together to facility women use of health facility delivery
	***Unintended-homebirth*** Network members intended to facilitate women’s facility delivery, but women had homebirths (n = 10)		Most women (n = 8) received advice to use facility-based pregnancy and or delivery care	Network members did not make arrangement in time to get women to a health facility for birth delivery
	***Homebirth–***Network members had no intention of facilitating women’s delivery in a health facility, so women had homebirths (n = 8)		Only 1 women received advice to use facility-based pregnancy (antenatal) care	Network members provided support oriented towards ensuring safe homebirth for women

#### Emotional support

We observed no difference in emotional support received by the three groups of women. Network members commonly expressed care and love by regularly visiting with women in order to enquire about their wellbeing. For example, one woman in the unintended-homebirth group described how her mother and MIL expressed care:

*M*: *…in what ways do you think Sarah (mother) was there for you and attentive to your needs when you were pregnant [waves]?*

*R*: *When I am not able to go visit her, she comes to visit me and asks about me*, *that made me know she loves me*

*M*: *what about Cecilia (MIL)?*

*R*: *Cecilia too shows me love [inaudible] so she chats with me about things that will bring laughter* [CR woman, Student, 19 yrs.].

This woman lived in the same house with her MIL and her mother lived nearby. Both network members visited with her daily during her pregnancy. She had a “*very close*” relationship with her mother and said of her MIL: “*we are free and open with each other*.” FGDs with MILs and IDIs with husbands further confirmed the emotionally supportive roles of these network members. Several husbands and MILs explained that due to their intimate relationships with the women, they listened and conversed with them about their pregnancy related concerns. A few women indicated that they did not have “*close*” relationships with their MILs, but most viewed their MILs as caring, and described their relationship as “*good*”.

#### Informational support

In FGDs with MILs and IDIs with husbands, it became clear that it is socially normative for in-laws, particularly MILs, mothers and grandmothers to advise women and provide suggestions on how to experience safe pregnancy and delivery. Generally women were advised on how to “*take care of myself well*” and “*eat well*,” i.e. healthy foods; and “*that I should not work too hard*” or “*not lift heavy things*,” as that will affect the baby.

Most women in the facility birth group (n = 11) and the unintended-homebirth group (n = 8) mentioned that advice and suggestions received from network members included regularly utilizing facility-based pregnancy and or delivery care. These network members lived in close proximity, and generally had good relationships, with the women. For example, a woman in the facility birth group who received support from her in-laws and friends mentioned:

*Respondent: Hamdia (brother-in-law’s wife)…and my husband. They told me to not work as hard as I used to because now that I am pregnant I need to be cautious of the kind of work that I do…Bintu (Friend) was also involved…if I was not feeling well, I would call her and tell her*. *She would then tell me to go to the hospital because that is where we will find out what is actually wrong with me …*

*Interviewer*: *it’s why I ask*, *how was your own experience* (*with father-in-law*)?

*Respondent*: *if you are sick and your husband is not there*, *your husband's father would say*, *“take her to the hospital*,*” and they (in-laws) will do that*. [NR woman, Farmer, 24 yrs.]

This woman interacted with her husband and in-laws daily because they lived in the same house. Her relationship with her husband’s family was generally “*good*,” as they regularly enquired about her wellbeing. The woman noted that she and her brother-in-law’s wife “*helped and advised*” each other. She also had regular phone conversations with her longtime friend who lived in another town, and they sometimes saw each other on market days.

Compared with the facility birth and unintended-homebirth groups of women, only one woman in the homebirth group mentioned that she received any advice related to seeking health facility care during pregnancy. The woman in the homebirth group (NR, Farmer, age unknown) explained that her co-wife, husband’s brother’s wife and aunt advised her to go to the hospital for care when she was feeling sick. None of her network members, however, advised her to give birth at a health facility.

#### Instrumental support

Labor and delivery narratives of women and husbands revealed differences in how instrumental support from network members impacted birth delivery location among the three groups of women. In the experience of women in the facility birth group, network members who lived in close proximity and had regular contact with the women and with each other collaborated to ensure women’s use of facility delivery. MILs confirmed in the FGDs that they involved other network members in women’s pregnancy and delivery care. One explained, “*When her husband is not there…you [MIL] would then talk to any family member available at that time*, *for that person to look for a motorbike*, *fuel it and take her to hospital*” (NR MIL). The pregnancy experience of one woman (CR, 24 yr. old unemployed) revealed that during her labor she first informed her MIL who in turn informed other network members. The woman’s father-in-law secured a ride from his brother to take her to the health facility, accompanied by her MIL and grandmother.

The 24 yr. old woman’s experience is analogous to that of other women in the first group. As the husband of another woman explained:

*Respondent: …I came to the house later and realized that she (wife) was in the room struggling. I asked her how she was and she said she was having stomach pains. Then I came out and told my mother who then asked me to go get my auntie. After my aunt come to see her (wife), she told us that my wife was in labor and that she should be taken to the clinic. So, I called my brother and we used his motorbike to transport my wife to the clinic. There she delivered [baby crying]*.

*Interviewer*: *so who assisted her (wife) through this process…?*

*Respondent*: *my auntie helped in supporting my wife to sit on the motorbike*. *My auntie sat at the back as we took my wife to the clinic*. [NR husband, Farmer, 27 yrs.]

Network members of the 27 yr. old husband’s wife lived nearby, frequently visited with her during her pregnancy, and thus were able to help her access and utilize facility delivery. Commonly, network members of the first group of women recognized the urgency of the women’s labor and readily mobilized resources to get them to a health facility for delivery. There was only one instance in which a woman (NR, Farmer, 30 yrs.) had a prolonged labor at home, for four days, before her husband’s younger brother and aunt assisted her to the hospital for delivery.

Similar to the facility birth group, network members of women in the unintended-homebirth group planned to assist the women’ use of facility birth. Yet, unlike the former, network members in the unintended-homebirth group tended to first seek the involvement of a traditional birth attendant (TBA) during women’s labor and did not make timely arrangements to transport women to a facility. The TBAs were involved in the process of deciding on whether it was time to send women to a health facility, which led delays in getting women to a health facility. For example, a NR woman (Farmer, 26 yrs.) explained that a TBA was initially called once she started experiencing labor pains: “…*when she [TBA] came*, *she suggested that I should be sent to Chamba hospital*. *They were preparing to send me*, *when I delivered*. Similarly, another woman mentioned that when she went into labor she informed her mother and they “*…started preparing for us to go the hospital but we were too late*, *as I delivered in the house*.” She explained that she first informed her mother and sister of her labor:

*Interviewer*: *what did your mother do when you told her?*

*Respondent*: *she told me to wait for a while because she was going call Esi Eyeh (Traditional Birth Attendant [TBA]) for her to come and check whether my pregnancy was due.*

*Interviewer*: *what did Effuah (sister) also do?*

*Respondent*: *she was praying for me that things would go well*. [CR woman, Trader, 20 yrs.]

The 20 yr. old woman lived with her mother and sister who were available to assist her in getting to a health facility for birth. However, they waited to find a TBA to examine her before preparing to go to the facility. Generally during women’s labor network members including MILs, co-wives or other elderly women (e.g. grandmother) in the women’s network were informed of the women’s labor. Although these members were available to assist the women, they waited to consult with a TBA or make certain of the woman’s labor before attempting to get women to a facility.

In very few exceptions the unintended-homebirth group of women gave birth at home shortly after going into labor, despite network members’ efforts to quickly make arrangements to get them to a health facility. For example, during her labor a woman (NR, Farmer, 20 yrs.) informed 3 of her brother-in-law’s wives who lived with her in the same house, and they in turn called in her “*close and trusted*” friend who lived nearby to assist her. She elaborated: *we were preparing to go to Gushegu (hospital) for the delivery and while he (husband) went out to look for a motor bike I gave birth in the house*.” A TBA assisted in the woman’s birth delivery, shortly after she went into labor.

The labor and delivery experiences of women in the homebirth group were dissimilar from women in the facility birth and unintended-homebirth groups in that women in the unintended-homebirth group indicated that their network members intended to help them experience safe labor and delivery at home. A husband described the labor experience of his wife:

*R: She was in pain for a while*, *so I just hanged around and monitored her condition here in the house. I kept moving around the house and praying that she delivers without any problem. They later told me she had delivered…*

*Facilitator*: *Ok [paused] so who assisted her through this process?*

*Respondent*: *There were two people who helped her…they were lakpeku (TBA) and Ayi (brother-in-law’s wife)* [NR husband, Farmer, 37 yrs.]

The 37 yr. old husband’s wife corroborated his account. She mentioned that her brother-in-law’s wife called a TBA to assist in her childbirth at home. She lived in the same house with her brother-in-law’s wife and they had a good relationship, as “*they advised each other on how to live with their husbands*.” Generally, the unintended-homebirth group of women indicated that network members relied on TBAs to assist in their delivery at labor onset. Network members made no plans to send women to a health facility. Instead, once women informed network members of their labor, a husband, MIL, bother-in-law’s wife would call in a TBA to assist in the women’s labor and delivery.

Despite these differences all three groups of women indicated that it was common practice for husbands, in-laws and blood-relatives to provide them with instrumental support. Across all three groups of women in-laws in the NR and female blood relatives in CR who lived in close proximity with women typically provided help with house chores–e.g. fetching water, washing and cooking. Network members who lived far and or had infrequent contact with women tended to send, or provide during their visits with women, money, food and items to help with pregnancy related care.

## Discussion

Overall, we found that social networks contribute in important ways to women’s use of facility-based pregnancy and delivery care. Different network members were involved in providing women with various kinds of social supports. We found differences in informational and instrumental support receive by the three groups of women. These women’s network support experiences highlighted ways in which network members were able to impact women’s place of childbirth.

One difference found among the three groups of women was in terms of informational support. Most women in the facility birth group and unintended-homebirth group, unlike those in the homebirth group, indicated that their network members advised them to utilize facility-based pregnancy and delivery care. Previous research conducted in rural Bangladesh and Western Kenya have similarly shown that through advice network members were able to inform women on their options for facility delivery and what to do to seek and access facility care [29, 42]. Moreover, studies in Low- and middle income countries (including El Salvador, Kenya, Bangladesh and Uganda) found that women who received advice from network members to seek facility-based care, and also instrumental support including money and transportation for pregnancy related care, help with house chores and food during pregnant, were more likely to have facility delivery compared with those who did not received such support [29, 31, 42–44].

During women’s birth labor, network members have to address challenges to facility delivery including distance, transportation and costs associated with delivery [2, 8, 9]. Delays in getting women to a health facility would prevent women from receiving appropriate labor care or assistance in managing any birth and labor complication [2]. These concerns were irrelevant for the facility birth group of women in our study, as their network members worked together to organize resources to support women’s access and use of facility birth. The collective action of network members and the sense of immediacy with which they sought care for women enabled women’s use of facility birth. This sense of urgency was, however, not observed in the unintended-homebirth group of women, since their network members sought the services of a TBA to ensure that the women were in labor before making preparation for the women to seek facility birth. Moreover, instrumental support for the homebirth group of women was oriented toward ensuring that they gave birth at home, as network members had no intention of getting the women to a health facility for delivery. These findings suggest that in order for social support to lead to facility birth network members’ attitudes and behaviors should reflect a normative belief in facility delivery. In terms of differences in how instrumental support impacted place of childbirth for the three groups of women, our findings suggest that in order for social support to lead to facility birth network members’ attitudes and behaviors should reflect a normative belief in facility delivery.

Perceived norms regarding the importance of health facility births are positively associated with health delivery [45–48]. Research in a number of African countries revealed that women whose husbands and MILs had positive perceptions about health facility birth were more likely to deliver in a health facility than those whose husbands and MILs did not have such perceptions [46, 48, 49]. This is because informational and instrumental support provided by these network members is often directed towards helping women to utilize facility-based pregnancy and delivery care. In our study all women in the facility birth group and most women in the unintended-homebirth group indicated that their network members intended to facilitate their use of facility birth, and moreover received informational (advice) and or instrumental support for facility birth. This finding contributes to growing evidence of a shift in social norms toward use of facility birth in Ghana [10, 30, 50, 51]. However, we note that network members of the homebirth group of women may not necessarily adhere to the normative belief regarding the importance of facility birth. As such, their support provision for women’s pregnancy care was not oriented toward facility birth.

Previous research examined birth location preferences of pregnant women, as well as the preference of various network members including husbands and mothers-in-law [34, 52]. The present study however, focused on the birth location intentions of network members as whole. We note that it may be truly challenging to differentiate the intentions of all members in a women’s network for a couple of reasons. First, network members may potentially have competing interest regarding the women’s place of birth. Second, it is socially desirable for women to give birth in health facilities. Conversely, our examination of network support provided for the women in our sample enabled us to determine the extent to which instrumental support received by the women reflected the intentions of their network members.

While there is a possibility that social network support may not directly translate to the Ghanaian context, earlier qualitative research studies on social support in Ghana have identified the importance of instrumental and emotional support for pregnant women’s use of health facility care and having a labor companion [30, 53]. Similar to these studies, we developed interview guides to examine study participant’s own understanding of social network structure and functions. This approach ensured that our questions were culturally sensitive and reflected the social context of women in the NR and CR. Ghanaian researchers translated the interview guides into the local languages of study participants and pilot tested the guides to ensure that the intended concepts and meanings were conveyed and understood.

A few limitations to our study are worth mentioning. Participants were interviewed in their native languages and the interviews were translated into English. Potentially, there may have been some translation errors and the nuance in meaning of some of the translations may have been lost. To maintain data integrity, we reviewed interview transcripts while listening to the original audio interviews to ensure accuracy in translation. As participants were interviewed after women’s childbirth, there was the possibility of recall bias. Response bias may have occurred in instances when research assistants interviewed participants whose gender is opposite theirs. Also, certain pregnancy and childbirth related issues are considered inappropriate topics of discussion between a man and woman who are not related. Hence, study participants may have provided socially desirable responses, and neglected to provide a full account of network members’ involvement in their pregnancy experiences. Research assistants were trained to use probing questions to ensure that participants were provided as much accurate and relevant information as possible.

## Conclusion

In addition to receiving social support during pregnancy from various members in their networks, most women in our study indicated that these members intended to facilitate their health facility delivery. Women who experienced facility delivery had network members who responded to labor onset with a sense of urgency by immediately mobilizing resources to facilitate women’s health facility delivery. From our findings, it is clear that network members can and do work together to provide women with needed support to overcome barriers to facility-based delivery care. Future public health education and maternal health interventions should highlight the critical role of network members in ensuring that pregnant women receive such care during labor.
